# Associations between social isolation, loneliness, and objective physical activity in older men and women

**DOI:** 10.1186/s12889-019-6424-y

**Published:** 2019-01-16

**Authors:** Stephanie Schrempft, Marta Jackowska, Mark Hamer, Andrew Steptoe

**Affiliations:** 1 Department of Psychology, University of Roehampton, London, SW15 5PJ UK; 2 School of Sport, Exercise and Health Sciences, Loughborough University, Loughborough, LE11 3TU UK; 3 Department of Behavioural Science and Health, University College London, 1-19 Torrington Place, London, WC1E 6BT UK

**Keywords:** Social isolation, Loneliness, Objective physical activity, Ageing, Older adults

## Abstract

**Background:**

The impact of social isolation and loneliness on health risk may be mediated by a combination of direct biological processes and lifestyle factors. This study tested the hypothesis that social isolation and loneliness are associated with less objective physical activity and more sedentary behavior in older adults.

**Methods:**

Wrist-mounted accelerometers were worn over 7 days by 267 community-based men (*n* = 136) and women (*n* = 131) aged 50–81 years (mean 66.01), taking part in the English Longitudinal Study of Ageing (ELSA; wave 6, 2012–13). Associations between social isolation or loneliness and objective activity were analyzed using linear regressions, with total activity counts and time spent in sedentary behavior and light and moderate/vigorous activity as the outcome variables. Social isolation and loneliness were assessed with standard questionnaires, and poor health, mobility limitations and depressive symptoms were included as covariates.

**Results:**

Total 24 h activity counts were lower in isolated compared with non-isolated respondents independently of gender, age, socioeconomic status, marital status, smoking, alcohol consumption, self-rated health, limiting longstanding illness, mobility limitations, depressive symptoms, and loneliness (β = − 0.130*, p =* 0.028). Time spent in sedentary behavior over the day and evening was greater in isolated participants (β = 0.143*, p =* 0.013), while light (β = − 0.143*, p =* 0.015) and moderate/vigorous (β = − 0.112*, p =* 0.051) physical activity were less frequent. Physical activity was greater on weekdays than weekend days, but associations with social isolation were similar. Loneliness was not associated with physical activity or sedentary behavior in multivariable analysis.

**Conclusions:**

These findings suggest that greater social isolation in older men and women is related to reduced everyday objective physical activity and greater sedentary time. Differences in physical activity may contribute to the increased risk of ill-health and poor wellbeing associated with isolation.

## Background

The extent to which individuals are interconnected and embedded in communities has a profound impact on health and longevity [1]. Social isolation – the absence of regular contact with family and friends and lack of involvement in social organizations; and loneliness – the subjective experience or perception of being isolated – are important concerns in the welfare of older people, as well as having health implications. Socially isolated individuals often experience feelings of loneliness, but social isolation and loneliness are often only weakly correlated [2, 3]. Some individuals may be content with having limited social contact, while others may have frequent social contact but feel lonely. Social isolation and loneliness have therefore been identified as distinct constructs, with potentially distinct pathways to disease [4].

Longitudinal studies have documented associations between both social isolation and loneliness and risk of mortality and the development of major chronic illnesses independently of other sociodemographic factors and pre-existing health conditions [5–8]. These associations have been corroborated by meta-analytic reviews, and appear not to be accounted for by publication bias [9, 10]. The search for mechanisms underlying these effects has focused on two primary pathways. The first is that direct psychobiological processes are responsible, with greater isolation and loneliness stimulating neuroendocrine dysregulation [11], disturbances in autonomic function and blood pressure control [12, 13], inflammatory responses [14, 15], and chronic allostatic load [16]. The second is that health behaviors contribute to increased health risk, with socially isolated and lonely individuals having less favorable lifestyles. Social relationships have been associated with not smoking, drinking moderately and with healthy diets [1, 17]. An analysis of UK Biobank indicated that health behavior accounted for more than 30% of the excess risk of mortality attributed to social isolation and loneliness over a 6.5 year follow-up period [18].

Links between social relationships and physical activity may be particularly important, since sustained physical activity is associated with a range of beneficial outcomes including reduced cardiovascular risk, reduced disability and frailty, beneficial metabolic profiles, greater independence, and quality of life [19]. There are several ways in which social relationships can promote physical activity. Friends and family might explicitly encourage physical activity, or they might have an implicit influence through their own behavior (also known as social control) [20]. The individual might also be more likely to engage in social events that are inherently active, whether it may be visiting friends and relatives, exercise classes, attending cultural events such as theater or concerts, or simply traveling outside of the home. The latter may be particularly relevant for older adults, as much of their physical activity accumulates through short trips out of the home.

There is evidence that social isolation in early life predicts future physical inactivity [21], but studies of older people, and those examining both social isolation and loneliness, remain sparse. A large study of older adults in Belgium found that more television viewing (a proxy for sedentary behavior) was related to limited social participation, limited contact with neighbors, and higher levels of loneliness [22]. In the English Longitudinal Study of Ageing (ELSA) [23], social isolation and loneliness were associated cross-sectionally with low levels of moderate and/or vigorous physical activity, though in longitudinal analysis over 10 years only isolation was related to physical inactivity [24]. Cross-sectional associations between both isolation and lonelienss and physical activity have been deescribed in the UK Biobank [25], while in contrast, no significant cross-sectional or longitudinal relationships between social networks and physical activity were observed in a Chicago-based community study, with only loneliness being associated with diminished odds of physical activity [26].

All these findings were based on self-reports of physical activity. Although such measures are important, physical activity tends to be systematically over-reported, with much greater amounts of activity being recalled than are evident in studies using objective measures [27, 28]. The inaccuracy of self-reports may be exaggerated among older people because of errors in recall, and because much activity is accumulated through everyday tasks and chores rather than defined episodes of formal exercise.

Accordingly, the present study investigated associations between social relationships, in the form of social isolation and loneliness, and accelerometer-based measures of physical activity in a sample of community-dwelling men and women aged 50 and older. It was hypothesized that social isolation and loneliness would be inversely related to physical activity counts both over weekdays and weekend days, and with greater sedentary behavior and less light or moderate/vigorous physical activity in waking hours, independently of age. Factors that might confound any association between social isolation/loneliness and physical activity were taken into account. The first was physical health, since poor health is associated with social isolation and loneliness [2, 29], and may also limit capacity for physical activity. Second, problems with mobility and impairments in activities of daily living (ADLs) may restrict social activity and increase feelings of loneliness, while also being linked with low physical activity [30]. Third, social isolation and loneliness are positively associated with depression at older ages [31], and reduced physical activity is characteristic of individuals with more depressive symptoms. Research also indicates that socioeconomic status partly explains links between social isolation/loneliness, disease risk [25], and mortality [18]; and socioeconomic status is associated with physical activity levels [32]. An association between social isolation/loneliness and physical inactivity could be secondary to any of these factors, therefore they were included in analyses.

## Methods

### Study population

The English Longitudinal Study of Ageing (ELSA) is a nationally representative panel study of people aged 50 years or older living in England [33]. Data are collected every two years using computer-assisted personal interviews and self-completion questionnaires, with home visits from a research nurse every four years for the collection of biomarkers. Participants give written informed consent, and the study protocol has been approved by the National Research Ethics Service. As part of wave 6 (2012–13), a random subsample (*n* = 499) was invited to wear an accelerometer for 8 days and complete a daily sleep log. Four hundred (80%) of those who were invited took part. But technical problems with some accelerometers (*n* = 28), insufficient wear time (*n* = 38) and loss of some sleep logs (n = 4) in transit reduced the sample size to 330. Of the 330 participants, only those with data on all the study variables were included in the analyses.

### Measures

#### Social isolation

The social isolation measure was created by assigning one point if the respondent had less than monthly contact (face-to-face, telephone, or written/e-mail) with each of children, other family members, and friends, and if they did not participate in organizations such as social clubs, religious groups, or committees. A similar index has been used in previous studies [7, 34]. Marital status was not included in this study because being unmarried does not necessarily imply social isolation, especially in the case of older adults who have been married and raised children, but whose spouse has recently died [4]. Scores could range from 0 to 4, with higher scores indicating greater social isolation. Because of the small sample size and skewed distribution (only 4% scored a 3 or 4 out of a maximum score of 4), participants were divided into those reporting no social isolation (score 0) and individuals reporting some isolation (score 1 or more) in the primary analyses.

#### Loneliness

Loneliness was assessed using the 3-item short form of the Revised UCLA (University of California, Los Angeles) Loneliness Scale [35], as used in previous studies [7, 23]. An example item is ‘How often do you feel you lack companionship?’ Items are scored on a three-point scale (1 = hardly ever, 3 = often) and summed to create an overall score, with higher scores indicating greater loneliness. Internal consistency was high in the present sample (Cronbach’s α = 0.87).

#### Objective physical activity

Participants wore a triaxial accelerometer (GeneActiv; Activinsights Ltd., Cambs, UK) on their non-dominant wrist for 8 consecutive 24-h days. The accelerometer was fitted in participants’ homes at the end of the nurse visit. They were also asked to complete a daily diary to report sleep and wake times and activities such as bicycling, and any periods of non-wear. Physical activity was sampled at 50 Hz and stored in gravity units (1 unit = 9.81 m/sec^2^). The first day was discarded, and data were extracted from midnight on day 2 until the end of day 8, leading to a maximum of 7 days of 24-h measurements. Periods of non-wear time were identified using algorithms from the GeneActiv software; only those with at least 95% wear time per day, and those who wore the device for at least one weekday and one weekend day, were included in the analysis. The data were converted into 1 min epochs and analyzed in two ways. First, the total counts of activity at any intensity were summed over 24 h for weekdays, weekend days, and the combination of week and weekend days. Second, the number of minutes per hour between 7:00 h and 22:00 h spent in sedentary activity, light activity and moderate / vigorous physical activity was calculated using validated cut-points [36]. Separate values were calculated for weekdays, weekend days, and the complete week.

#### Heath and mobility impairment

Self-rated health was measured using a well-established single item known to predict disease outcomes and mortality [37]. Participants were asked to rate their health into five categories: excellent, very good, good, fair, and poor. To assess the presence of chronic illness, participants were asked if they had any long-standing illness, and if so, whether it limited their ability to look after themselves or participate in activities. Limiting longstanding illness is strongly associated with the presence of chronic illnesses at older ages, and is a valid health measure for surveys [38, 39]. Mobility was assessed by asking about 10 activities involving leg mobility and arm function (e.g. walking 100 yards, reaching or extending your arms above shoulder level), and was modeled as a continuous variable.

#### Depressive symptoms

Depressive symptoms were measured using the 8-item Centre for Epidemiologic Studies Depression Scale (CES-D) [40]. As in previous research, the CES-D question on loneliness was not included in the total scale score [23]. A score of ≥3 was used to indicate significant symptomatology, as in previous studies [41].

#### Socioeconomic factors

Total (non-pension) wealth was calculated net of debt, and included financial wealth, the value of any property, business assets, and physical wealth such as jewelry and artwork [42]. Age-related quintiles of wealth were used in the analyses. Educational attainment was categorized as no formal qualifications, intermediate (high school education), and higher education (college education).

#### Other variables

Marital status was classified into married/cohabiting and other. Age was classified into 4 categories: 50–59 years, 60–69 years, 70–79 years, and 80 or older. Smoking status (3 categories: non-smoker, ex-smoker, current smoker) and alcohol consumption (5 or more times per week (daily) vs. less than daily) were also assessed since these have been associated with activity levels [43, 44].

### Statistical analysis

The characteristics of isolated and not isolated groups were compared using Χ^2^ for categorical variables and t-tests for continuously distributed variables. Results are presented as N (percentage) and means ± standard deviation. T-tests and Pearson’s correlations were used to assess associations between loneliness and other characteristics. Differences in activity counts between weekdays and weekend days were analyzed using repeated measures analysis of variance with day (weekday, weekend) as the within-person factor and social isolation as the between-person factor. The associations between social isolation or loneliness and objective activity were analyzed using combined weekday and weekend data. Separate regressions were carried out on total activity counts, and the mean number of minutes per hour spent in sedentary behavior and light and moderate/vigorous activity. The moderate/vigorous physical activity data were significantly skewed; therefore a log transformation was used. Four models were evaluated in each analysis. Model 1 presents unadjusted associations between social isolation or loneliness and activity; model 2 was additionally adjusted for gender, age, educational attainment, non-pension wealth, marital status, smoking, alcohol consumption, limiting longstanding illness, number of mobility impairments, and self-rated health; depressive symptoms were added in model 3; loneliness or social isolation was added in model 4. In the analysis of total activity counts, accelerometer wear time was also included as a covariate. Additional adjustment for employment status did not change the pattern of results so this was not included in the final models. Results are presented as standardized regression coefficients (β) with standard errors (SE) for social isolation and loneliness. Variance inflation factor values were calculated for all regression models to assess multicollinearity, and all were within an acceptable range.

## Results

Accelerometer data were obtained from 330 individuals, and usable information over at least two days was available from 316. The number of days of wear averaged 5.79 ± 1.31. Of these participants, 292 completed the measures of social isolation and loneliness, and there was missing information on covariates for 25 individuals. The analytic sample was therefore 267 (107 isolated and 160 not isolated respondents). Participants ranged in age from 50 to 81 years, averaging 66.01 (standard deviation 7.81) years. As shown in Table 1, there were no significant differences between isolation groups in gender distribution, age, education, wealth, smoking, alcohol consumption, self-rated health, limiting longstanding illness, number of mobility impairments, depressive symptoms, loneliness score, or number of days of accelerometer wear. However, isolated participants were less likely to be married (*p* = 0.05). Loneliness scores were higher among women (t (248.05) = − 3.52, *p* = 0.001), unmarried participants (t (265) = − 4.69, *p* < 0.001), current smokers (t (46.82) = − 2.30, *p* = 0.026), those who consumed alcohol less than 5 times per week (t (79.38) = 2.64, *p* = 0.010), those with elevated depressive symptoms (t (47.12) = − 4.77, *p* < 0.001), and those with a limiting longstanding illness (t (145.10) = − 2.54, *p* = 0.012). Loneliness was also positively correlated with the number of mobility impairments (r = 0.29, p < 0.001) and self-rated health (1 = excellent, 5 = poor; r = 0.16, *p* = 0.007), and negatively correlated with age categories (r = − 0.13, *p* = 0.04) and wealth quintiles (r = − 0.24, *p* < 0.001). There was no association with the number of days of accelerometer wear.

**Table 1:** Characteristics of socially isolated and not isolated groups (n (%) unless stated otherwise; *N* = 267^a^)

	Isolated (*n* = 107)	Not isolated (*n* = 160)	P difference
Sex			
Male	60 (56.1)	76 (47.5)	0.17
Female	47 (43.9)	84 (52.5)	
Age, years			
50–59	21 (19.6)	37 (23.1)	0.92
60–69	46 (43.0)	67 (41.9)	
70–79	36 (33.6)	50 (31.3)	
≥ 80	4 (3.7)	6 (3.8)	
Education			
No qualifications	52 (48.6)	55 (34.4)	0.07
Intermediate	23 (21.5)	44 (27.5)	
Higher education	32 (29.9)	61 (38.1)	
Wealth quintile			
1 (lowest)	26 (24.3)	28 (17.5)	0.21
2	27 (25.2)	33 (20.6)	
3	21 (19.6)	28 (17.5)	
4	21 (19.6)	39 (24.4)	
5 (highest)	12 (11.2)	32 (20.0)	
Married	56 (52.3)	103 (64.4)	0.05
Smoking status			
Non-smoker	80 (74.8)	132 (82.5)	0.19
Former smoker	9 (8.4)	6 (3.8)	
Current smoker	18 (16.8)	22 (13.8)	
Alcohol consumption			
< 5 times per week	87 (81.3)	131 (81.9)	0.91
5–7 times per week	20 (18.7)	29 (18.1)	
Self-rated health			
Excellent	9 (8.4)	27 (16.9)	0.33
Very good	31 (29.0)	46 (28.7)	
Good	40 (37.4)	52 (32.5)	
Fair	22 (20.6)	26 (16.3)	
Poor	5 (4.7)	9 (5.6)	
Longstanding limiting illness	42 (39.3)	45 (28.1)	0.06
No. of mobility impairments, mean SD	2.07 ± 2.4	1.52 ± 2.3	0.06
Depressive symptoms ≥3	16 (15.0)	25 (15.6)	0.88
Loneliness score, mean SD	1.47 ± 0.6	1.38 ± 0.5	0.21
Days of accelerometer wear, mean SD	5.84 ± 1.3	5.80 ± 1.3	0.80

^a^Subsample from the English Longitudinal Study of Ageing, wave 6 [2012–13]

### Objective physical activity on weekdays and weekend days

**Fig. 1:**
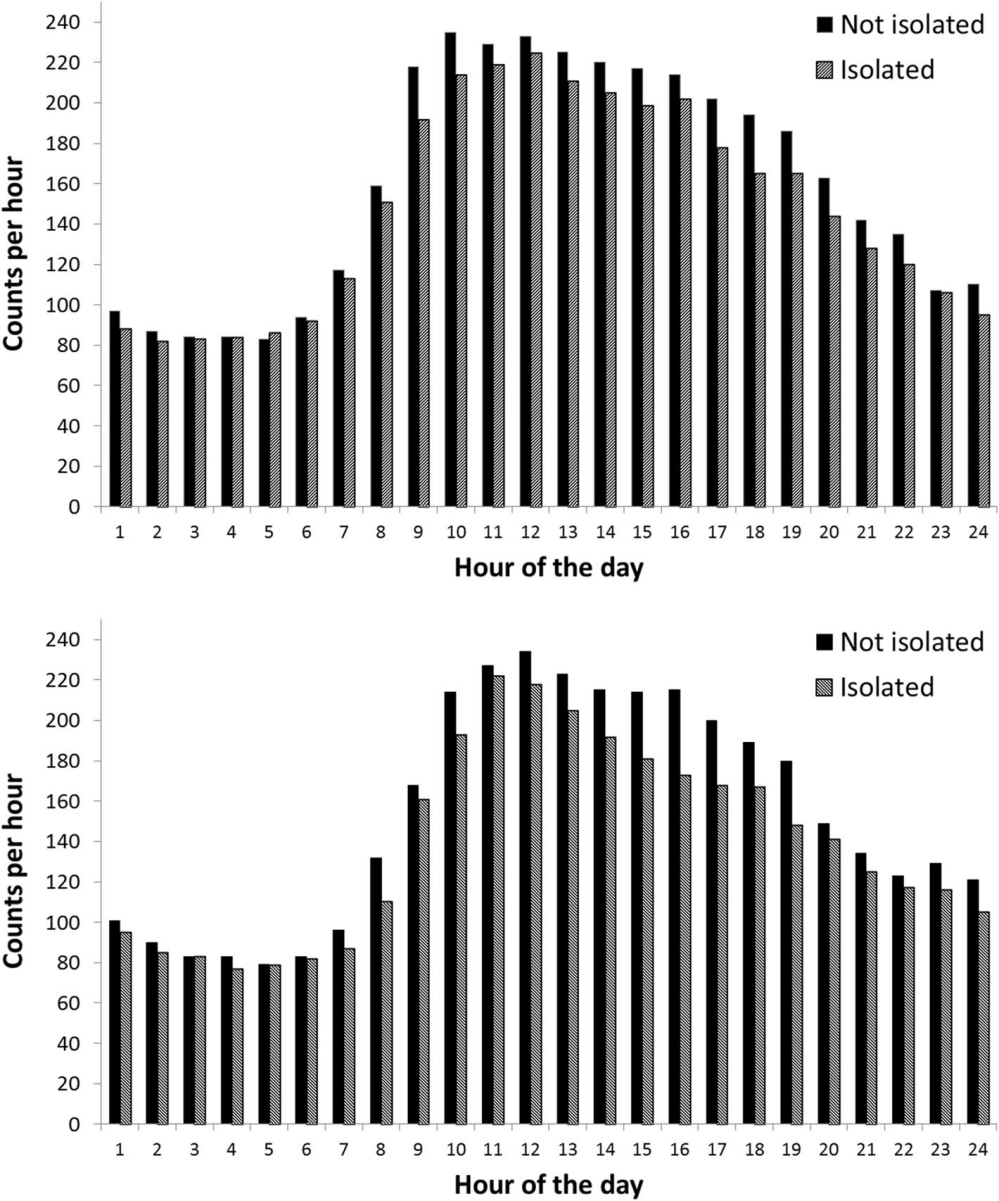
Mean accelerometer counts per minute avearged over each hour of the 24 h period. Data for weekdays are shown in the upper panel; data for weekend days are shown in the lower panel. Isolated participants are in hatched bars and not isolated in solid bars

The mean activity count per hour in isolated and not isolated participants are summarized in Fig. 1 for weekdays and weekend days. The relatively high counts at night are the result of the greater sensitivity of the wrist-worn GeneActiv compared with conventional accelerometers. Counts began to increase on average around 7:00–8:00 h and peak at 10:00–13:00 h before declining into the evening. Average counts were consistently lower in the isolated participants during the day time, converging with those of the not-isolated group in the evening. Repeated measures analysis of variance identified main effects for social isolation (*p* = 0.003) and day (*p* < 0.001), but no interaction between the two. Twenty-four-hour activity counts were lower in the isolated participants, and higher on weekdays compared with weekend days. Analyses of different physical activity categories also showed no interaction between isolation and day of the week. For convenience, the multiple variable regressions were therefore carried out on activity values aggregated across week and weekend days. In univariate analyses, total activity counts were inversely correlated with age, self-rated health, limiting longstanding illness, and mobility impairment (*r* = − 0.18 to − 0.32, *p ≤* 0.004), and positively correlated with wealth (r = 0.16, *p* = 0.010). Similar patterns were observed for light and moderate/vigorous activity over the day, while positive associations were recorded for time spent in sedentary behavior.

### Social isolation and objective physical activity

The regressions of physical activity on social isolation are summarized in Table 2. The analysis of total activity counts showed a significant negative association with social isolation (model 1) which was attenuated when covariates were added in model 2. The β coefficient barely changed with additional adjustment for depressive symptoms (model 3) and loneliness (model 4). The final model accounted for 19.2% of the variance in activity counts. Apart from social isolation, the independent predictors of activity counts in model 4 were age (β = − 0.212, *p* = 0.001) and self-rated health (β = − 0.286, *p* < 0.001).

**Table 2:** Associations between social isolation and objective physical activity (*N* = 267^a^)

	Social isolation unadjusted (Model 1)		Social isolation adjusted for gender, age, educational attainment, non-pension wealth, marital status, smoking, alcohol consumption, limiting illness, mobility impairment, and self-rated health (Model 2)		As in Model 2, but additionally adjusted for depressive symptoms (Model 3)		As in Model 3, but additionally adjusted for loneliness (Model 4)	
*Outcome variable:*	β (S.E.)^b^	P	β (S.E.)	P	β (S.E.)	P	β (S.E.)	P
Total counts (24 h)	− 0.172 (0.061)^c^	0.005	−0.135 (0.059)^c^	0.022	−0.133 (0.059)^c^	0.024	−0.130 (0.059)	0.028
Waking time (7:00–22:00 h)								
Sedentary activity	0.202 (0.060)	0.001	0.149 (0.057)	0.010	0.145 (0.057)	0.011	0.143 (0.057)	0.013
Light physical activity	−0.206 (0.060)	0.001	−0.149 (0.059)	0.012	−0.145 (0.058)	0.014	−0.143 (0.059)	0.015
MV physical activity	−0.153 (0.062)	0.014	−0.119 (0.057)	0.038	−0.116 (0.057)	0.044	−0.112 (0.057)	0.051

^a^Subsample from the English Longitudinal Study of Ageing, wave 6 [2012–13]

^b^Standardized regression coefficients (β) and standard error in parentheses

^c^Additionally adjusted for total accelerometer wear time

Social isolation was positively associated with sedentary activity over the waking period; the β coefficient for isolation was 0.143 (*p* = 0.013) in the fully adjusted model, which accounted for 23.3% of the variance in sedentary activity. The analyses of light and moderate/vigorous physical activity showed negative associations with social isolation (β = − 0.143; − 0.116) (the latter was borderline significant, see model 4).

**Fig. 2:**
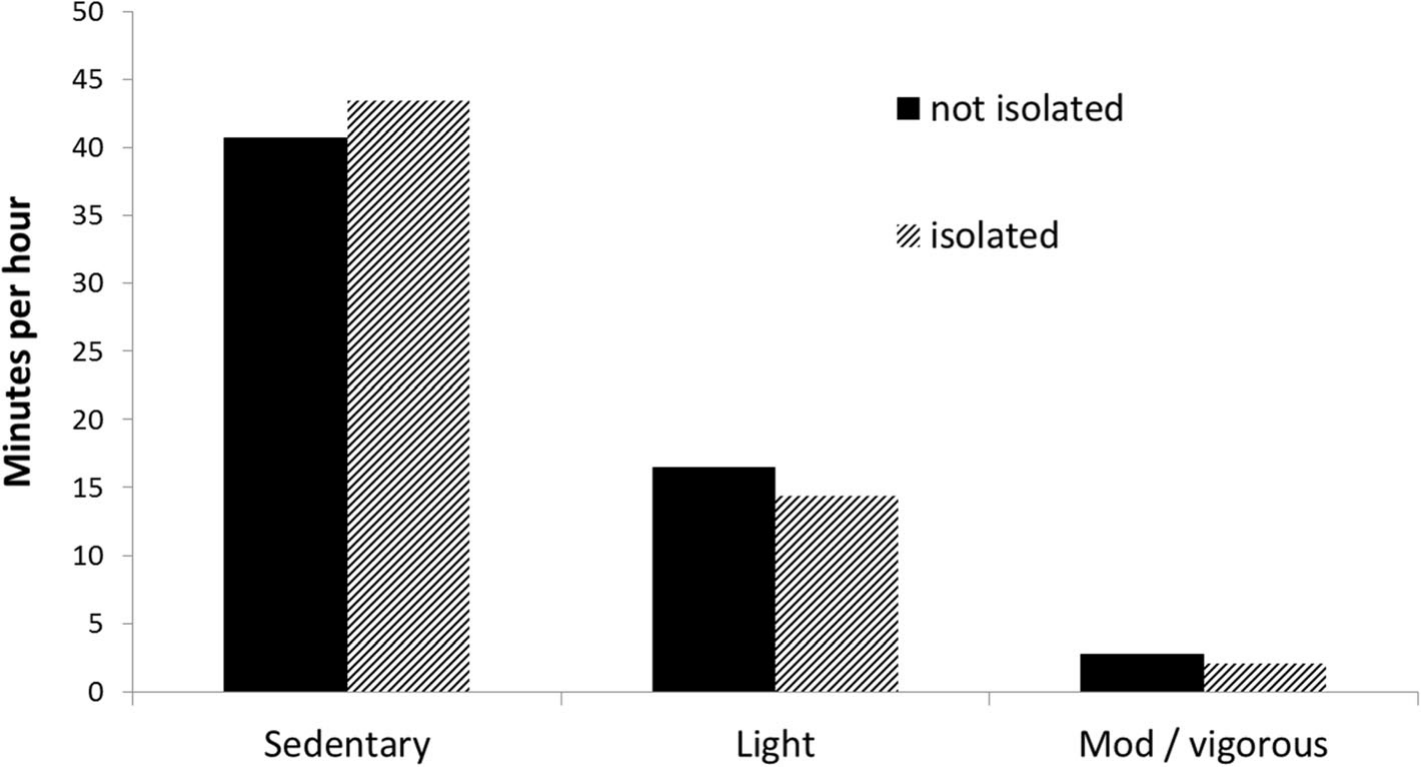
Average number of mintues per hour spent in sedentary behavior, light and moderate/vigorous physical activity. Isolated participants are in hatched bars and not isolated in solid bars

The average number of mintues per hour between 07:00 h and 22:00 h spent in sedentary behavior, light and moderate/vigorous physical activity (aggregated across week and weekdays) in isolated and not isolated participants are shown in Fig. 2.

### Loneliness and objective physical activity

The regressions of physical activity on loneliness are summarized in Table 3. The analysis of total activity counts showed a significant negative association with loneliness (model 1), but this was no longer significant when covariates were added in model 2. Sedentary, light, and moderate/vigorous physical activity were not associated with loneliness.

**Table 3:** Associations between loneliness and objective physical activity (*N*=267^a^)

	Loneliness unadjusted (Model 1)		Loneliness adjusted for gender, age, educational attainment, non-pension wealth, marital status, smoking, alcohol consumption, limiting illness, mobility impairment, and self-rated health (Model 2)		As in Model 2, but additionally adjusted for depressive symptoms (Model 3)		As in Model 3, but additionally adjusted for social isolation (Model 4)	
*Outcome variable:*	β (S.E.)^b^	P	β (S.E.)	P	β (S.E.)	P	β (S.E.)	P
Total counts (24 h)	−0.121 (0.061)^c^	0.048	−0.064 (0.065)^c^	0.332	−0.087 (0.068)^c^	0.199	−0.081 (0.067)	0.232
Waking time (7:00–22:00 h)								
Sedentary activity	0.106 (0.061)	0.084	0.040 (0.064)	0.529	0.079 (0.066)	0.233	0.072 (0.065)	0.275
Light physical activity	−0.072 (0.061)	0.239	−0.019 (0.066)	0.770	−0.058 (0.068)	0.392	−0.051 (0.067)	0.450
MV physical activity	−0.120 (0.063)	0.055	−0.044 (0.065)	0.493	−0.071 (0.067)	0.286	−0.062 (0.067)	0.347

^a^Subsample from the English Longitudinal Study of Ageing, wave 6 [2012–13]

^b^Standardized regression coefficients (β) and standard error in parentheses

^c^Additionally adjusted for total accelerometer wear time

## Discussion

This study investigated the association between social relationships, in the form of social isolation and loneliness, and objectively measured physical activity in a community sample of men and women age 50 to 81 years old. Social isolation was associated with lower total activity counts over 24 h, and with more time spent in sedentary behavior and less time in light and moderate/vigorous physical activity over the day and evening. These relationships were apparent on week and weekend days, and were independent of gender, age, socioeconomic status, marital status, indicators of health, mobility impairment, depressive symptoms, and loneliness. Loneliness was not associated with physical activity or sedentary behavior once gender, age, and other covariates had been taken into account.

The study of social isolation and loneliness, and how they might impact health is an important research endeavor. In the USA, social network size is thought to have declined over recent decades, with reductions in kin and non-kin confidants and fewer community and neighborhood connections [45], although there are suggestions that these changes may result from methodological limitations [46]. In the UK, around one third of people aged 65 and older live alone, and up to 20% have less than monthly contact with friends and family [47]. Prevalence of loneliness has remained relatively stable among older adults over the past decade; although around one third report feeling lonely [48].

This study utilized an accelerometer-based measure of activity rather than self-report in order to provide objective evidence of links with social relationships. Self-report measures are the bedrock of most observational epidemiology relating activity with health outcomes. Nevertheless, changes in norms with age and disability of what constitutes vigorous activity, problems in recall, and inaccurate completion of measures in which moderate or high intensity activities predominate can limit the accuracy of self-reports in older adults [49]. The accelerometers used in this study were strapped to the wrist rather than waist in order to provide information over the night as well as daytime.

The physical activity levels in this population were very low, with an average of 2.4 min per hour in moderate/vigorous activity, and 15.5 min per hour in light activity over the day and evening. Respondents therefore spent an average 42.1 (70.2%) minutes per hour in sedentary behavior. A recent systematic review concluded that across 22 studies of people aged ≥60 years, 65–80% of the waking day is spent in sedentary behavior [50]. The values in this study fall at the lower end of this distribution, partly because respondents aged 50–59 years were included. Physical activity levels were significantly higher on weekdays than weekend days, although differences were relatively small. This difference has not always been found in accelerometer studies of older people, particularly when respondents were on average in their 70s and 80s, compared with the younger participants in the present sample [51, 52].

Social isolation was associated with greater sedentary behavior and lower levels of light and moderate/vigorous physical activity. These findings are consistent with a possible role of physical activity in the health risk associated with social isolation. The differences were not large, but if small differences in physical activity persist in the everyday lives of more isolated individuals, they will accumulate over time and contribute to a raised likelihood of chronic disease and disability in older people.

Previous findings on the association between loneliness and self-reported physical activity have been mixed, with several studies finding no association [53–55]. In the large-scale study of more than 3000 older adults, social isolation but not loneliness was consistently associated with a greater risk of being inactive over a 10 year period [24]. The findings of this study are in line with the notion that social isolation has stronger links with health behaviors than loneliness does. A possible explanation is that socially isolated individuals lack positive social influences from family members and friends, so are less likely to be exposed to (and therefore influenced by) both injunctive norms (what significant others say or want an individual to do) and descriptive norms (how significant others actually behave), which are predictive of health behaviors [56, 57].

A cross-sectional association between social isolation and physical activity does not necessarily indicate a direct link, since the association could be secondary to other factors that are related to social behavior and physical activity. Poor self-rated health, limiting longstanding illness, and mobility limitations were related to reduced activity and greater sedentary behavior in bivariate analyses. However, the multivariable regressions suggest that these factors play a moderate role in explaining the association, since the β coefficient fell from − 0.172 to − 0.135 when these factors had been included in the models.

The cross-sectional nature of the study also prevents causal conclusions from being drawn. It is possible that greater social isolation leads to reduced activity, but it is also plausible that less active people withdraw from social connections. However, the fact that the measure of social isolation included telephone and electronic communication as well as face to face meetings means that reduced physical activity would not preclude social contacts. It has also been shown that much of the physical activity of older people accumulates through trips out of the home which can have both practical and social functions.

The meaningfulness of the social isolation measure has been established, since it has been shown to predict mortality and other health outcomes [7, 58]. However, this study compared isolated with not isolated individuals, and the effects may be stronger in a sample with a greater range of social integration. The measure of social isolation was comprehensive in that it considered contacts with friends, family, and children, in addition to civic participation, but it did not take network size into account. Some aspects of social contact might have stronger links with physical activity than others, and this is a topic for future work. Another issue for future research would be to examine potential interactive influences of social and loneliness on physical activity, but this would require a larger sample size than the one analyzed here [59].

A strength of this study is that it was embedded within a well-characterized longitudinal cohort study.

## Conclusions

These findings suggest that greater social isolation in older men and women is related to reduced everyday physical activity and greater sedentary time. Differences in physical activity may contribute to the increased health risks associated with isolation.

## Abbreviations

ADLs: Activities of daily living
CES-D: Centre for Epidemiologic Studies Depression Scale
ELSA: English Longitudinal Study of Ageing
UCLA: University of California, Los Angeles
